# The Genetics of Cerebellar Structure and Associations With Cognitive Performance: A Twin Magnetic Resonance Imaging Study

**DOI:** 10.1002/hbm.70300

**Published:** 2025-08-06

**Authors:** Gretchen Lutz, Simon Smerconish, David Roalf, Michael C. Neale, J. Eric Schmitt

**Affiliations:** ^1^ Department of Radiology, Division of Neuroradiology Hospital of the University of Pennsylvania Philadelphia Pennsylvania USA; ^2^ Department of Psychiatry, Brain Behavior Laboratory Hospital of the University of Pennsylvania Philadelphia Pennsylvania USA; ^3^ Departments of Psychiatry and Genetics Virginia Institute for Psychiatric and Behavioral Genetics, Virginia Commonwealth University Richmond Virginia USA

**Keywords:** cerebellum, cognition, genetics, MRI, twin

## Abstract

The cerebellum, traditionally associated with motor control, is increasingly recognized for its involvement in higher‐order cognitive functions. However, the role of cerebellar subregions in cognition remains underexplored, as are the roles of genetic factors on cerebellar structure and brain‐behavioral associations. The primary goal of this study was to investigate the relationship between cerebellar subregion volumes and cognitive performance. A secondary aim was to quantify the genetic contributions to cerebellar structure and determine the degree to which any brain‐behavior associations were genetically mediated. 3T anatomic MRI data from *N* = 932 typically developing individuals from the Human Connectome Project were used for this study. Twenty‐seven cerebellar regions of interest (ROIs) were automatically parcellated using *CerebNet*. Three additional lobar‐level ROIs were derived from smaller measures. Nine functional domains (six cognitive and three motor) related to known or suspected cerebellar function were selected. Linear regression analyses were conducted to identify correlations between cerebellar volumes and cognitive outcomes, adjusting for age, sex, and overall brain volume. Univariate and bivariate quantitative genetic modeling was then performed in OpenMx. There were numerous statistically significant phenotypic associations between cognitive measures and cerebellar lobar and lobular volumes, particularly in the IPL, AL, bilateral cortices, left lobule V, right lobule VI, vermis, and vermis lobule VIII, each meeting the threshold of *p* < 0.02 across at least four out of nine cognitive domains. The vermis and vermis lobule VIII were of particular note, showing even stronger associations (*p* < 0.0009 across three domains). Cognitive measures were modestly heritable, and cerebellar ROIs were highly heritable. Quantitative genetic models suggested that brain–behavior associations are largely driven by shared environmental factors. Our findings identify novel associations between specific cerebellar subregions and cognitive performance, highlighting the vermis as a critical structure. We also provide a detailed map of the quantitative genetics of human cerebellar structure. Future studies are warranted.

## Graphical abstract

This study is a comprehensive examination of the genetics of cerebellar structure and its cognitive correlates using genetically‐informative Human Connectome Project data and structural equation modeling. Individual differences in most cerebellar subregions are dominated by genetic variation. There are weak but statistically significant associations with several cognitive measures, with the observed phenotypic associations largely driven by familial effects.

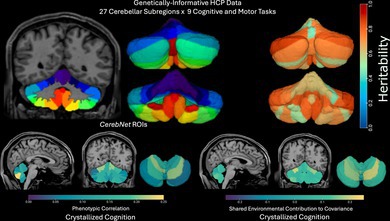

## 1 Introduction

Although considered exclusively a motor structure for nearly 200 years, the cerebellum is increasingly recognized for its involvement in numerous higher brain functions (Schmahmann 2019; Stoodley and Schmahmann 2009). We now understand that the involvement of the cerebellum in motor tasks is particularly associated with its small anterior lobe (AL), comprised of lobules I–V (Jacobi et al. 2021). In contrast, the larger superior posterior lobe (SPL) has been found to play a significant role in the coordination of non‐motor tasks including cognition, emotion, and language; lesions to SPL influence affect and thinking (Buckner 2013; Moberget et al. 2014; Rapoport et al. 2000). The specific functional neuroanatomy of the inferior posterior lobe (IPL) is even less well understood than other cerebellar lobes, but seems to be important to cognition, as lesions in this region can cause cerebellar cognitive affective syndrome (Stoodley and Schmahmann 2009). More generally, the preponderance of evidence suggests that the cerebellum is responsible for the coordination of multiple types of information and is therefore involved in many complex tasks, with some regional specificity based on connectivity with different cortical areas (Buckner et al. 2011; Xue et al. 2021). Neuroimaging research has also implicated the cerebellum in the pathogenesis of several psychiatric, neurological, and genetic disorders including autism spectrum disorder (ASD), attention deficit hyperactivity disorder (ADHD), Alzheimer Disease, Parkinson Disease, and the 22q11.1 deletion syndrome (Mapelli et al. 2022; Schmitt et al. 2023; Singh‐Bains et al. 2019; Yin et al. 2021; Yu et al. 2023).

Despite our improved awareness of the importance of the cerebellum in higher cognitive function, there remain relatively few studies of regional variability in cerebellar structure and cognition in typically‐developing populations. This limitation is partly owed to historical challenges in obtaining suitably‐sized neuroimaging samples to explore subtle brain‐behavior associations, as well as a lack of computational tools to parcellate the cerebellum both reliably and with high spatial resolution. However, the advent of both large, high‐quality, publicly‐available datasets and automated cerebellar parcellation algorithms now makes comprehensive investigations of cerebellar functional anatomy possible.

The primary goal of the current study was to investigate the regional specificity of cerebellar structure on cognitive and motor functions in the typically developing (TD) population. In order to accomplish this goal, we leverage the high‐resolution structural MRI data available in the Human Connectome Project (HCP). We also take advantage of the development of *CerebNet*, a novel machine learning algorithm for cerebellar parcellation which enables measurement of individual cerebellar lobule volumes and derived lobar measures. Additionally, we exploit the genetically informative nature of HCP data to explore the relative contributions of genetics and environmental factors to the structure of cerebellar regions of interest (ROIs).

## 2 Methods

The study utilized data from the Human Connectome Project (HCP) S1200 release, a comprehensive study investigating brain‐behavior relationships in young, healthy adults (Van Essen et al. 2013). Acquired data included structural MRI, task‐based functional MRI (fMRI), and a broad array of standardized cognitive variables. A subset of the data was acquired on monozygotic (MZ) and dizygotic (DZ) twins (Table 1). The current study included a total of *N* = 932 individuals. The study was approved by the Institutional Review Board of the Hospital of the University of Pennsylvania.

**TABLE 1:** Demographic characteristics of the sample and summary statistics for behavioral measures.

	MZ	DZ	Singletons	Total	ICC^a^
*N*	242	134	556	932	
Age (years)	29.3 (3.3)	29.3 (3.5)	28.4 (3.9)	28.8 (3.6)	
Sex	100 M (41%)	48 M (36%)	271 M (49%)	419 M (45%)	
	142 F (59%)	86 F (64%)	285 F (51%)	513 F (55%)	
CogTotalComp	112.2 (20.9)	115.8 (21.9)	112.2 (20.2)	112.7 (20.7)	0.87
CogCrystalComp	108.4 (17.1)	110.9 (18.2)	109.7 (16.6)	109.5 (16.9)	0.88
CogFluidComp	105.6 (17.2)	107.7 (16.7)	104.6 (17.5)	105.3 (17.3)	0.63
CogEarlyComp	106.2 (16.6)	108.0 (15.9)	106.1 (16.0)	106.4 (16.2)	0.59
WMTaskAcc	85.9 (10.7)	87.2 (9.8)	86.6 (9.1)	86.5 (9.6)	0.43
LangTaskAcc	88.3 (7.7)	90.1 (6.8)	89.0 (6.8)	88.9 (7.1)	0.69
Strength	102.1 (19.1)	102.2 (18.1)	104.1 (20.7)	103.3 (19.9)	0.87
Endurance	109.5 (14.3)	107.3 (14.0)	106.6 (14.1)	107.5 (14.2)	0.83
Dexterity	100.4 (9.4)	100.9 (9.5)	99.8 (10.0)	100.1 (9.8)	0.45

^a^ Test–retest reliability estimates from a *N* = 46 HCP subsample.

### 2.1 Image Acquisition and Processing

**FIGURE 1:**
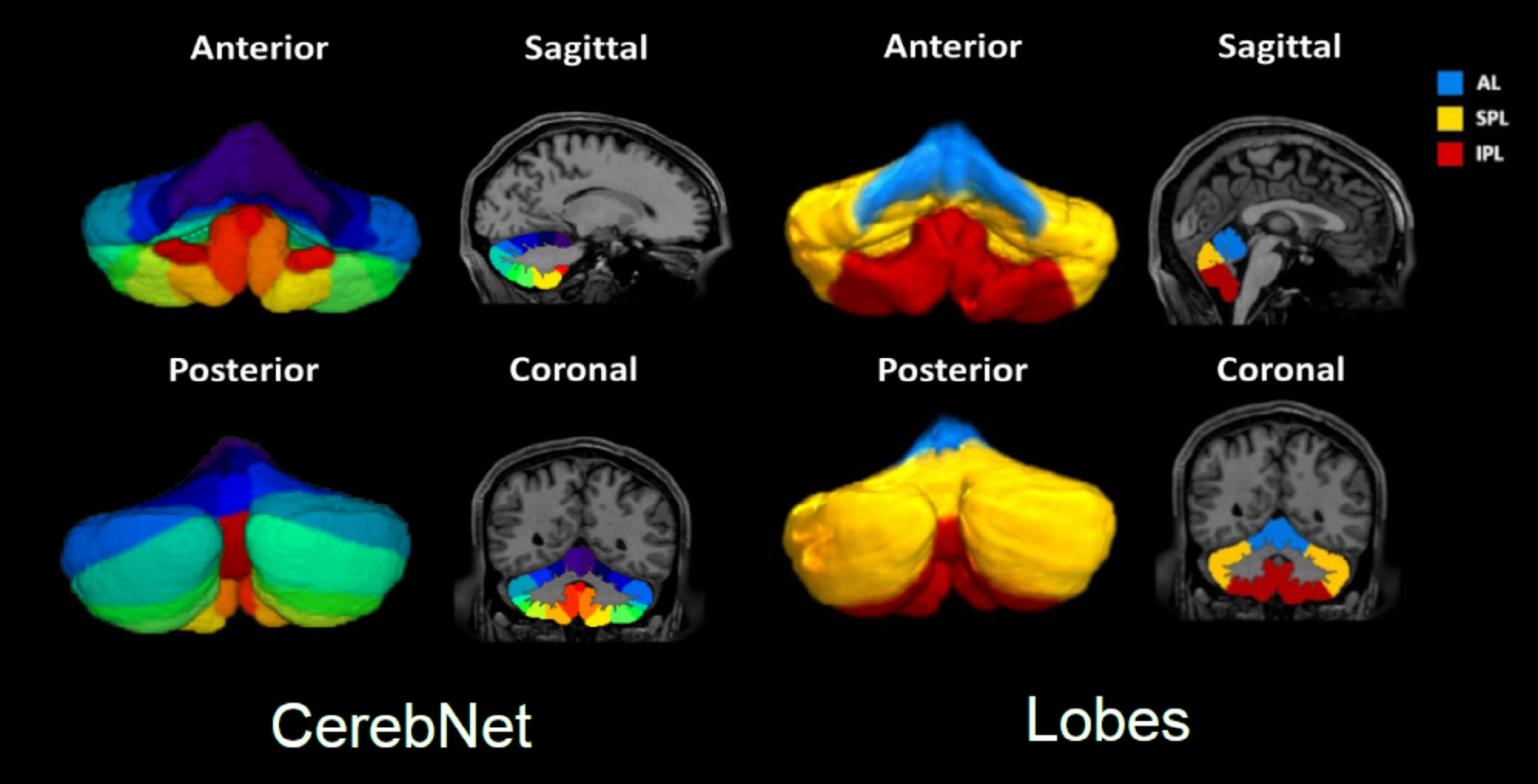
*CerebNet* ROIs (left) and derived cerebellar lobes (right).

All data were acquired on the same 3 Tesla Siemens Connectome scanner. The image acquisition protocol included high‐resolution T1 MPRAGE (TR = 2400 ms; TE 2.14 ms; flip angle = 8°; FOV = 224 × 224 mm^3^; voxel size 0.7 mm isotropic). In order to generate volumetric measures of cerebellar lobes, native T1‐weighted images were processed using the *CerebNet* pipeline (Faber et al. 2022). This pipeline first extracts the total cerebellum using *FastSurfer* segmentation and isolates cerebellar gray and white matter (Henschel et al. 2020). A U‐Net‐based Convolutional Neural Network (CNN) then models data from three orthogonal planes based on the atlas of Schmahmann (Schmahmann et al. 1999). The segmentation included 27 subsegments (10 hemispheric and 1 white matter for each hemisphere and 5 vermis; See Figure 1 and Table 2). This processing pipeline has a high accuracy, with average Dice coefficients of 0.87. Each subject's HCP segmentation was visually inspected by a board‐certified neuroradiologist (JES) with over 20 years of experience in quantitative neuroimaging. In addition to lobular measurements, aggregate lobar measurements were obtained by combining the following regions (Mankiw et al. 2017): AL—lobules I–V, SPL—lobules VI, Crus I and II, lobule VIIB; IPL—lobules VIIIA, VIIIB, IX.

**TABLE 2:** Volumes (mm^3^) for *CerebNet* ROIs in HCP data.

ROI	Mean	SD	Median
Left cerebellum cortex	50294.20	5079.79	49889.75
Left cerebellum white matter	13124.25	1470.86	13102.43
Right cerebellum cortex	50449.35	5203.37	50118.67
Right cerebellum white matter	13229.86	1498.39	13180.65
Total vermis	5547.65	640.50	5533.52
Left lobules I–IV	3155.33	462.65	3134.79
Left lobule V	3429.21	508.23	3413.22
Left lobule VI	7482.64	1129.54	7364.72
Left crus I	11855.26	1618.97	11834.56
Left crus II	7877.19	1231.52	7813.62
Left lobule VIIb	4729.25	694.35	4652.29
Left lobule VIIIa	4910.43	887.58	4824.13
Left lobule VIIIb	3372.82	563.28	3342.71
Left lobule IX	2914.22	551.78	2891.43
Left lobule X	567.86	82.55	564.84
Right lobules I‐IV	3154.25	452.95	3145.30
Right lobule V	3203.31	471.59	3161.67
Right lobule VI	7523.64	1181.30	7442.26
Right crus I	12257.07	1725.69	12191.94
Right crus II	8207.97	1210.89	8158.58
Right lobule VIIb	4359.36	643.27	4309.42
Right lobule VIIIa	4769.01	859.89	4712.80
Right lobule VIIIb	3445.03	562.69	3422.89
Right lobule IX	2959.41	568.51	2942.80
Right lobule X	570.31	85.63	564.30
Vermis VI	1652.61	240.79	1647.15
Vermis VII	717.76	137.35	704.89
Vermis VIII	1951.73	298.76	1939.92
Vermis IX	856.36	115.52	851.44
Vermis X	369.20	59.03	365.35

### 2.2 Cognitive and Motor Testing

Cognitive and motor function was assessed using a battery of standardized tests that spanned six cognitive domains and three motor domains, largely selected from available HCP data based on a hypothesized association with cerebellar function. Cognition was assessed using the NIH Toolbox (Heaton et al. 2014). Cognitive assessment included age‐adjusted composite measures of fluid cognition (CogFluidComp), crystallized cognition (CogCrystalComp), and total cognition (CogTotalComp; a combination of fluid and crystalized scores). HCP data also include assessment using the Early Childhood Composite test (CogEarlyComp), which is an average of standard scores from flanker, picture vocabulary, card sort, and picture sequence memory tasks. We also examined two in‐scanner behavioral measures obtained during task‐based fMRI data acquisition. Working memory task accuracy (WM_Task_Acc) measured global performance from a standard 2‐back (vs 0‐back) task that included multiple categories of stimuli (faces, body parts, tools, places). Language Task Accuracy (Language_Task_Acc) measured average accuracy from an experiment that assessed both language ability (via comprehension of short stories) and a control adaptive math task (Binder et al. 2011). Motor measures were also selected from the NIH Toolbox and included age‐adjusted endurance, dexterity, and strength (Reuben et al. 2013). Briefly, the endurance task measures the distance that a subject is able to walk in 2 min, the dexterity task measures the time it takes for a subject to place and remove 9 pegs in a pegboard, and the strength task measures hand grip strength. In general, test–retest reliability for NIH Toolbox motor tasks has been reported as *r* = 0.92 for endurance, *r* = 0.85 for dexterity, and *r* = 0.92 for strength (Reuben et al. 2013). Test–retest reliability estimates for HCP data specifically (in a subsample of *N* = 46 MZ twins) are provided in Table 1.

### 2.3 Statistical Analyses

Cerebellar volumes and behavioral variables were imported into the R statistical environment for analysis (R Core Team 2022). Basic statistics were calculated, and ROI volumes were assessed for normality. Linear regression models were then used to assess brain‐behavior associations between the volumes of the 32 cerebellar ROIs and the 9 cognitive and motor domains using age, sex, and total brain volume as covariates. Standardized beta weights were calculated to estimate effect sizes of cognitive‐cerebellar associations. Control for multiple testing was performed using false discovery rate (FDR) (Genovese et al. 2002).

### 2.4 Quantitative Genetic Modeling

Prior to genetic analyses, the data were reformatted from individual‐wise to family‐wise records. Genetic modeling was then performed in OpenMx, a structural equation modeling (SEM) package fully integrated into the R environment (Boker et al. 2011; Neale et al. 2016). Univariate ACE SEMs were constructed to decompose phenotypic variance into additive genetic (A), shared environmental (C) and unique environmental (E) components based on differences in correlational patterns between relatives, primarily between monozygotic (MZ) and dizygotic (DZ) twins (Schmitt et al. 2021). To maximize power, an extended twin design (ETD) was used, which incorporates all available familial relationships in the HCP dataset, including nontwin families and siblings of twins (Posthuma and Boomsma 2000). All models controlled for the mean effects of sex and age. Serial univariate ACE models were fitted to data on all cerebellar ROIs, both with and total brain volume (TBV) as a global covariate. Univariate models were also constructed for our nine behavioral measures. Optimum model fit was determined via maximum likelihood. Proportions of variance were calculated by dividing individual variance components by the total phenotypic variance, *V*. For example, the heritabilty (${\hat{a}}^{2}$) is the additive genetic variance divided by the total phenotypic variance, A/V. Likelihood‐based 95% confidence intervals were also calculated for all variance components (Neale and Miller 1997). To test for the statistical significance of individual variance components, the fit of our full models were compared to nested submodels that removed subcomponents from the model (e.g., CE vs. ACE to test for additive genetic effects); in likelihood‐based SEM, differences in fit generally follow *c* ^2^ with degrees of freedom equal to the difference in free parameters. However, due to boundary constraints, differences between univariate ACE and nested submodels follows a 50:50 mixture *c* ^2^ with 0° and 1° of freedom (Dominicus et al. 2006).

To assess the underlying influences on intra‐cerebellar and cerebellar‐behavioral associations, genetically informative bivariate Cholesky decompositions were used to decompose phenotypic covariance (Schmitt et al. 2021). These models are similar to two univariate ACE models run in parallel, except that they also allow for covariation between the observed variables via A, C, and E latent factors. From these models, the additive genetic covariance can be calculated. Genetic correlations (*r*_*G*_) were then calculated by standardizing the genetic covariance matrix, mathematically defined as:

$$r_{x,y}=\frac{A_{\mathrm{xy}}}{\sqrt{A_{x}*A_{y}}\;}$$

Where $A_{\left\{\mathrm{xy}\right\}}$ is an off‐diagonal element of A, and $A_{x}$ and $A_{y}$ are the corresponding diagonal elements. Correlations for other variance components were calculated similarly. Given that correlations based on variance components can be deceptively large when proportional variance is small, we also calculated the genetic contributions to phenotypic covariance (pcor_A_). This measure accounts for the proportional genetic variance (i.e., the heritability) of each variable in the bivariate model:

$${\text{pcor}}_{A}=\sqrt{{\hat{a_{\mathrm{Var}1}}}^{2}}*r_{G}*\sqrt{{\hat{a_{\mathrm{Var}2}}}^{2}}$$

This metric also has its own disadvantages, for example it is not mathematically bounded to 0–1, but it can be considered complementary to the genetic correlation since it provides an estimate of the importance of genetic effects on total phenotypic variability (de Vries et al. 2021). Contributions of environmental variance components to the phenotypic covariance were estimated similarly. As with univariate models, the statistical significance of covariance can be calculated by comparing the full models to nested submodels where specific covariance paths are removed. For all hypothesis tests, post hoc corrections for multiple comparisons were applied using FDR.

All corrections used the Benjamini‐Hochberg algorithm from the ‘*p*.adjust’ function in base R. Our analyses included a total of 1305 hypothesis tests (288 phenotypic brain‐behavior associations, 27 on the quantitative genetics of cognitive and motor measures, 180 from univariate models of cognitive and cerebellar measures, and 810 bivariate quantitative genetic brain‐behavioral associations). Multiple testing correction was performed for each analysis separately. In order to perform a more stringent control for multiple testing, FDR correction was repeated on all *p* values in the study simultaneously, with overall similar statistical inferences; these results are provided as Supporting Information.

## 3 Results

### 3.1 Cross‐Trait Phenotypic Associations

**FIGURE 2:**
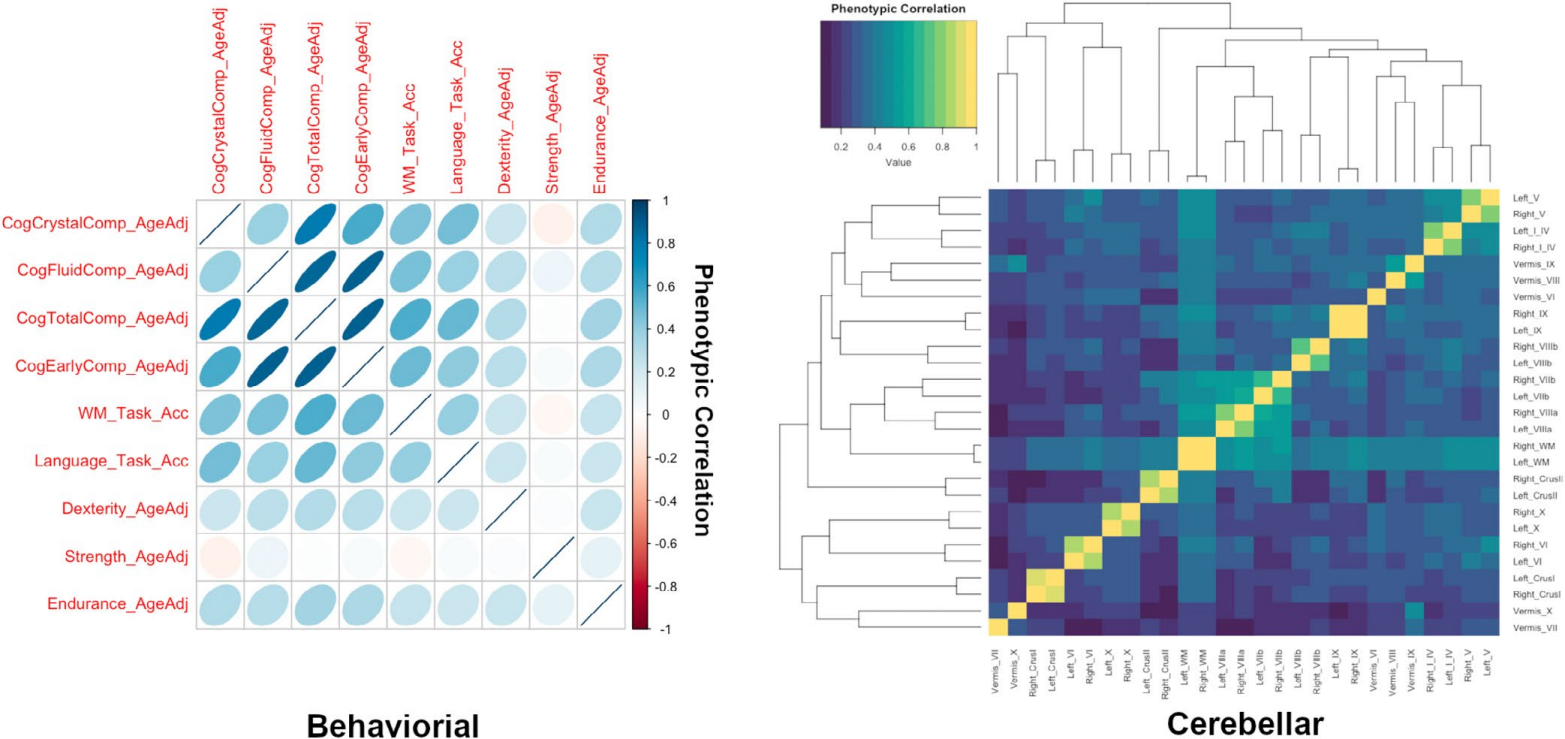
Phenotypic correlations for behavioral (left) and cerebellar (right) measures. For cognitive measures, stronger correlations are increasingly ovoid in shape. Cerebellar volumes are reordered based on hierarchical clustering.

Figures 2 and S1 summarize cross‐trait phenotypic correlations. The correlation between fluid and crystallized intelligence was modest (0.38), as were correlations between working memory and language/math accuracy and measures of cognition, which ranged from ~0.40–0.60. There also were moderate correlations between cognitive measures and two of the three motor measures, namely Dexterity and Endurance; Strength was effectively uncorrelated with the behavioral measures. Many intra‐cerebellar correlations were extremely high, particularly between contralateral homologues, which often approached unity. Higher correlations were seen between lobules within the same lobe (particularly AL), as well as between all cerebellar ROIs and the cerebellar white matter. High correlations were also observed between several vermis segments.

**FIGURE 3:**
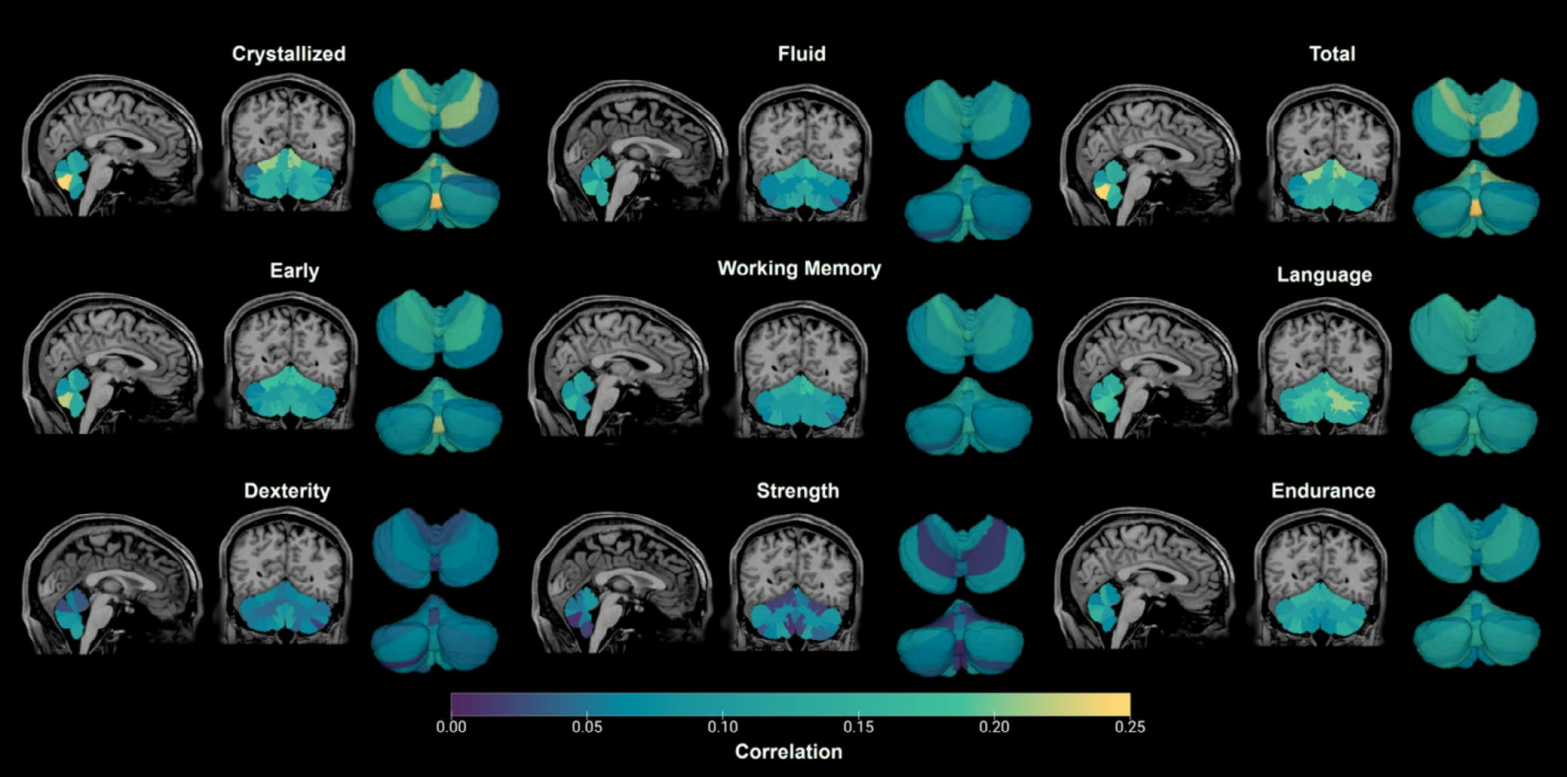
Brain‐behavior phenotypic correlations for lobular ROIs and 9 cognitive and motor measures.

**FIGURE 4:**
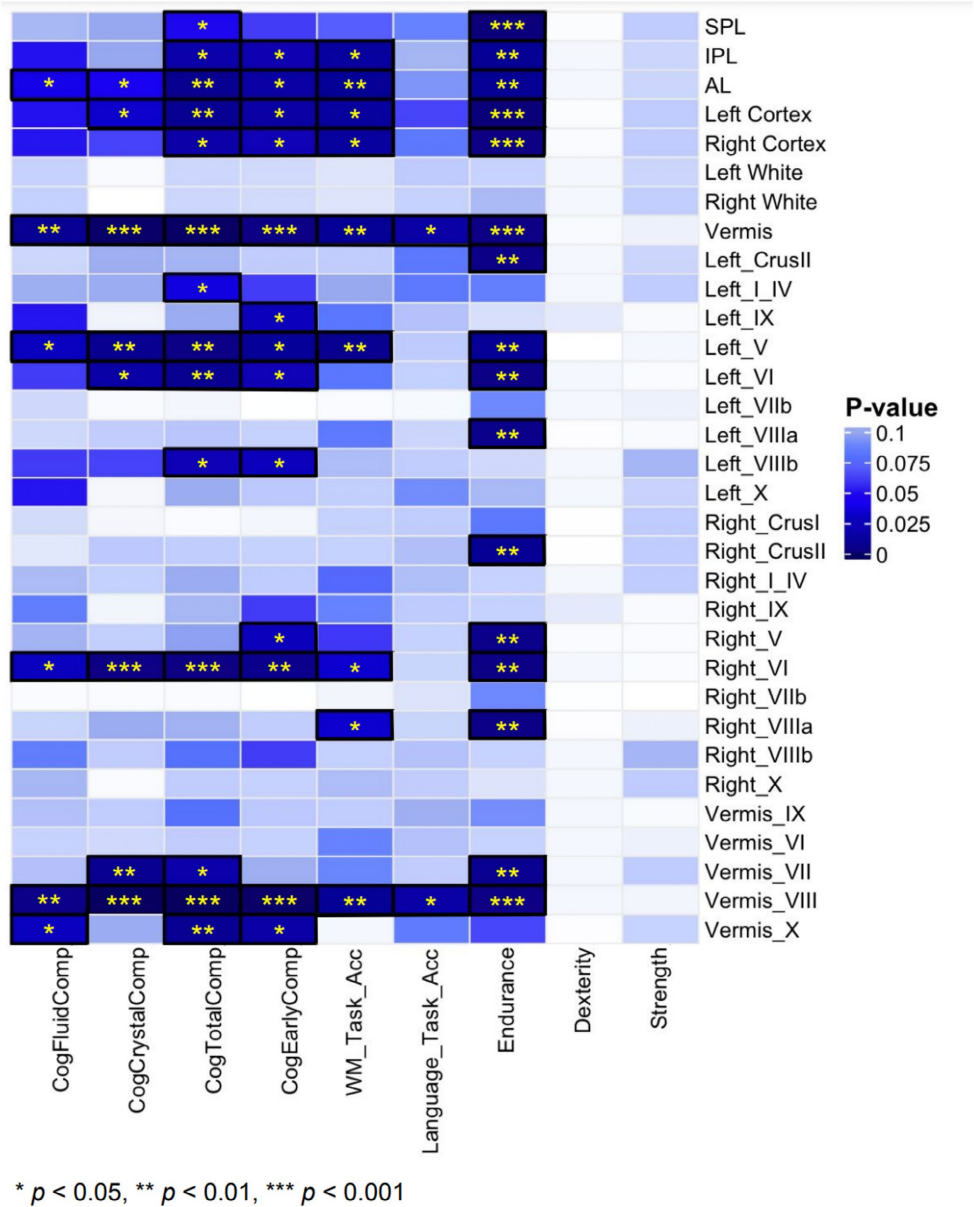
Statistical significance of cerebellar‐cognition associations. The heatmap shows FDR‐corrected *p* values, with darker colors showing stronger significance.

Phenotypic correlations between cerebellar ROIs and behavior scores were substantially less pronounced compared to the intra‐cerebellar and intra‐behavioral correlations (Figure 3). The strongest associations were seen between segment VIII of the vermis and both Total and Crystallized Cognition, although the magnitude of the association was small (*r* = 0.26). However, there were nevertheless statistically significant associations with multiple ROIs, particularly with the vermis (both whole and segmented), global hemispheric measures of the cerebellar cortex bilaterally, and right VI (Figure 4, Table S1). Statistically significant lobes/lobules had small but positive relationships with cognitive measures (Figure S2, Table S2), where increased volumes predicted improved performance in the corresponding cognitive domains. Notable subregions (those with three or more significant correlations) included the IPL, AL, left and right cortices, left V, left VI, right VI, vermis, and vermis VIII. Total vermis and vermis VIII were particularly significant across all measures excluding dexterity and strength, with *p* values ≤ 0.01.

### 3.2 Quantitative Genetic Analysis

**FIGURE 5:**
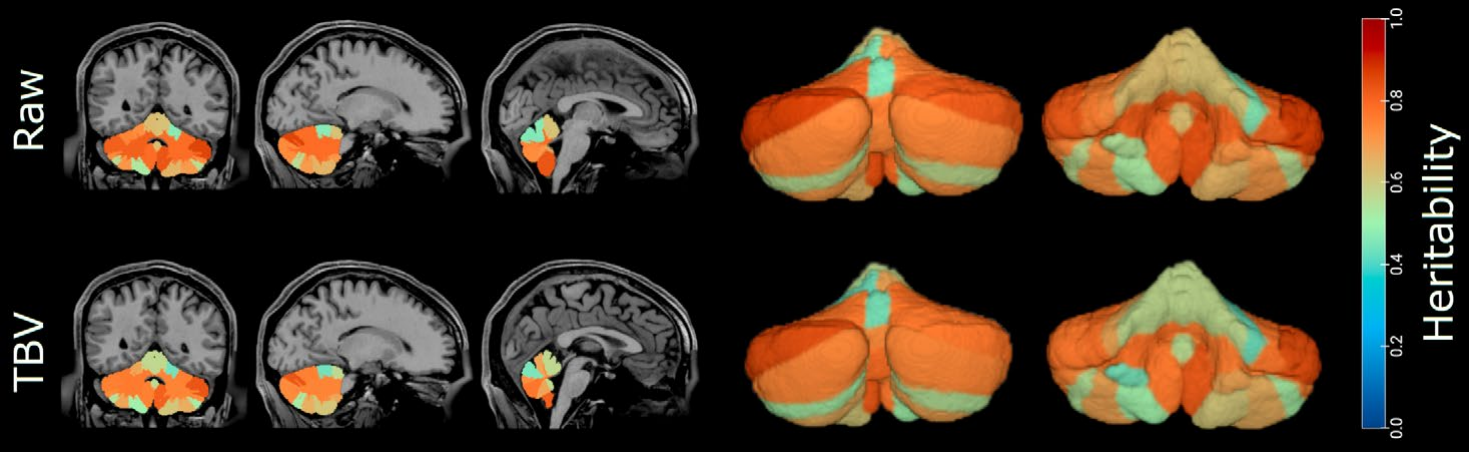
Heritability map of cerebellar ROIs from models both without and including a TBV covariate (TBV). All models included age and sex covariates.

Behavioral measures were modestly heritable (Table 3), with the strongest genetic effects seen for Crystalized Cognition (0.39), Endurance (0.43), and the language task (0.43). The shared environment also had a substantial influence on all cognitive measures and working memory (> 0.36), explaining more than a third of the phenotypic variance. Most cerebellar regions were highly heritable, with point estimates ranging from 0.47–0.86, and with most ROIs having more than half of their phenotypic variance attributable to additive genetic factors (Tables 4 and S3, Figure 5). In contrast, the role of the shared environment on cerebellar structure appeared minimal (0.00–0.23). Although using TBV as a covariate generally decreased heritability estimates slightly, the effect was small (Tables 4 and S4, Figure 5). After correcting for multiple testing, genetic effects were highly significant regardless of whether TBV was included as a covariate (*p* value < 0.0023), while shared environmental effects were not significant for any ROI for either model.

**TABLE 3:** Univariate variance components analysis results for nine cognitive‐behavioral measures.

Measure	${\hat{a}}^{2}$	${\hat{c}}^{2}$	${\hat{e}}^{2}$	*p*.A	*p*.C	*p*.AC
Crystalized cognition	0.39 [0.24 0.54]	0.39 [0.25 0.51]	0.22 [0.17 0.22]	< 0.0001	< 0.0001	< 0.0001
Fluid cognition	0.13 [0.00 0.37]	0.40 [0.07 0.39]	0.46 [0.39 0.64]	0.3581	< 0.0001	< 0.0001
Total cognition	0.26 [0.05 0.45]	0.42 [0.27 0.56]	0.32 [0.25 0.42]	0.0353	< 0.0001	< 0.0001
Early cognition	0.13 [0.00 0.37]	0.40 [0.22 0.54]	0.46 [0.36 0.58]	0.3581	< 0.0001	< 0.0001
Working memory	0.00 [0.00 0.20]	0.36 [0.25 0.44]	0.64 [0.56 0.61]	1.0000	< 0.0001	< 0.0001
Language	0.43 [0.15 0.58]	0.04 [0.00 0.24]	0.52 [0.42 0.65]	0.0105	0.7633	< 0.0001
Dexterity	0.19 [0.00 0.43]	0.10 [0.00 0.34]	0.72 [0.57 0.86]	0.3581	0.5158	< 0.0001
Strength	0.26 [0.00 0.52]	0.14 [0.00 0.34]	0.59 [0.47 0.74]	0.1537	0.2394	< 0.0001
Endurance	0.43 [0.19 0.54]	0.00 [0.00 0.27]	0.57 [0.46 0.70]	0.0072	1.0000	< 0.0001

*Note:* Proportional variance components are provided, as well as 95% confidence intervals (brackets) and FDR‐corrected *p* values testing for genetic (A), shared environmental (C), and familial (AC) effects.

**TABLE 4:** Univariate variance components analysis results for cerebellar ROIs.

ROI	${\hat{a}}^{2}$	${\hat{c}}^{2}$	${\hat{e}}^{2}$	*p*.A	*p*.C	*p*.AC	${\hat{a}}^{2}$	${\hat{c}}^{2}$	${\hat{e}}^{2}$	*p*.A	*p*.C	*p*.AC
Left_Cerebellum_Cortex	0.84	0.04	0.13	< 0.0001	1.0000	< 0.0001	0.76	0.08	0.16	< 0.0001	1.0000	< 0.0001
Left_Cerebellum_White_Matter	0.79	0.00	0.21	< 0.0001	1.0000	< 0.0001	0.74	0.00	0.26	< 0.0001	1.0000	< 0.0001
Right_Cerebellum_White_Matter	0.81	0.00	0.19	< 0.0001	1.0000	< 0.0001	0.76	0.00	0.24	< 0.0001	1.0000	< 0.0001
Right_Cerebellum_Cortex	0.78	0.06	0.16	< 0.0001	1.0000	< 0.0001	0.73	0.10	0.17	< 0.0001	0.8537	< 0.0001
Cbm_Left_I_IV	0.62	0.00	0.38	< 0.0001	1.0000	< 0.0001	0.57	0.00	0.43	0.0001	1.0000	< 0.0001
Cbm_Right_I_IV	0.63	0.02	0.35	< 0.0001	1.0000	< 0.0001	0.57	0.05	0.38	< 0.0001	1.0000	< 0.0001
Cbm_Left_V	0.47	0.13	0.40	0.0006	0.6448	< 0.0001	0.44	0.10	0.46	0.0023	1.0000	< 0.0001
Cbm_Right_V	0.72	0.00	0.28	< 0.0001	1.0000	< 0.0001	0.68	0.00	0.32	< 0.0001	1.0000	< 0.0001
Cbm_Left_VI	0.79	0.02	0.20	< 0.0001	1.0000	< 0.0001	0.75	0.03	0.23	< 0.0001	1.0000	< 0.0001
Cbm_Vermis_VI	0.46	0.23	0.31	< 0.0001	0.0520	< 0.0001	0.45	0.24	0.31	< 0.0001	0.0292	< 0.0001
Cbm_Right_VI	0.74	0.07	0.19	< 0.0001	1.0000	< 0.0001	0.76	0.04	0.20	< 0.0001	1.0000	< 0.0001
Cbm_Left_CrusI	0.86	0.00	0.14	< 0.0001	1.0000	< 0.0001	0.83	0.00	0.17	< 0.0001	1.0000	< 0.0001
Cbm_Right_CrusI	0.81	0.00	0.19	< 0.0001	1.0000	< 0.0001	0.75	0.04	0.21	< 0.0001	1.0000	< 0.0001
Cbm_Left_CrusII	0.73	0.00	0.27	< 0.0001	1.0000	< 0.0001	0.73	0.00	0.27	< 0.0001	1.0000	< 0.0001
Cbm_Right_CrusII	0.73	0.00	0.27	< 0.0001	1.0000	< 0.0001	0.73	0.00	0.27	< 0.0001	1.0000	< 0.0001
Cbm_Left_VIIb	0.56	0.00	0.44	< 0.0001	1.0000	< 0.0001	0.52	0.00	0.48	< 0.0001	1.0000	< 0.0001
Cbm_Right_VIIb	0.57	0.00	0.43	< 0.0001	1.0000	< 0.0001	0.55	0.00	0.45	< 0.0001	1.0000	< 0.0001
Cbm_Left_VIIIa	0.70	0.00	0.30	< 0.0001	1.0000	< 0.0001	0.66	0.00	0.34	< 0.0001	1.0000	< 0.0001
Cbm_Right_VIIIa	0.71	0.00	0.29	< 0.0001	1.0000	< 0.0001	0.68	0.00	0.32	< 0.0001	1.0000	< 0.0001
Cbm_Left_VIIIb	0.65	0.00	0.35	< 0.0001	1.0000	< 0.0001	0.61	0.00	0.39	< 0.0001	1.0000	< 0.0001
Cbm_Right_VIIIb	0.53	0.09	0.38	0.0003	1.0000	< 0.0001	0.51	0.07	0.41	0.0005	1.0000	< 0.0001
Cbm_Left_IX	0.83	0.00	0.17	< 0.0001	1.0000	< 0.0001	0.81	0.01	0.18	< 0.0001	1.0000	< 0.0001
Cbm_Vermis_IX	0.70	0.04	0.26	< 0.0001	1.0000	< 0.0001	0.68	0.00	0.32	< 0.0001	1.0000	< 0.0001
Cbm_Right_IX	0.79	0.00	0.21	< 0.0001	1.0000	< 0.0001	0.75	0.04	0.21	< 0.0001	1.0000	< 0.0001
Cbm_Left_X	0.71	0.03	0.25	< 0.0001	1.0000	< 0.0001	0.68	0.05	0.27	< 0.0001	1.0000	< 0.0001
Cbm_Vermis_X	0.63	0.00	0.37	< 0.0001	1.0000	< 0.0001	0.56	0.00	0.44	< 0.0001	1.0000	< 0.0001
Cbm_Right_X	0.55	0.14	0.30	< 0.0001	0.4820	< 0.0001	0.45	0.22	0.34	0.0014	0.0854	< 0.0001
Cbm_Vermis_VII	0.74	0.00	0.26	< 0.0001	1.0000	< 0.0001	0.72	0.00	0.28	< 0.0001	1.0000	< 0.0001
Cbm_Vermis_VIII	0.73	0.07	0.20	< 0.0001	1.0000	< 0.0001	0.77	0.00	0.23	< 0.0001	1.0000	< 0.0001
Cbm_Vermis	0.64	0.16	0.20	< 0.0001	0.2629	< 0.0001	0.63	0.12	0.25	< 0.0001	0.7830	< 0.0001

*Note:* Proportional variance components are provided, as are FDR‐corrected *p* values testing for genetic (A), shared environmental (C), and familial (AC) effects. Results from models either without (left) or including (right) TBV as a covariate are provided. 95% confidence intervals are provided as Supporting Information.

**FIGURE 6:**
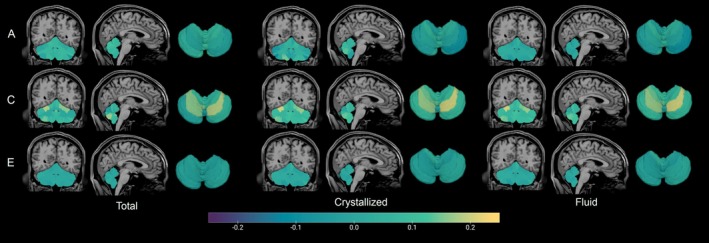
Contribution to the phenotypic covariance between measures of intelligence and cerebellar volumes for genetic (A), shared environmental (C) and unique environmental (E) variance components.

**FIGURE 7:**
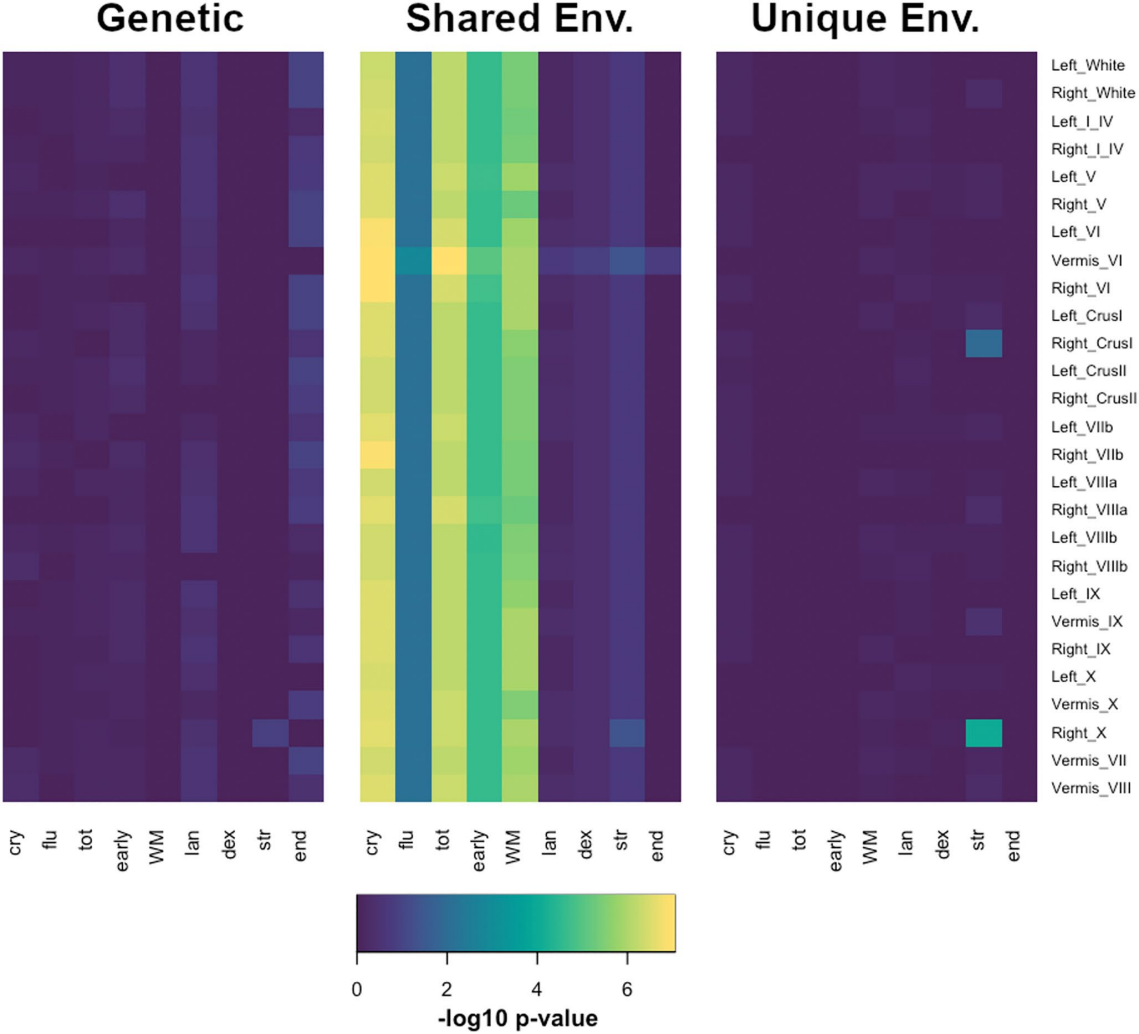
Statistical significance of brain‐behavioral covariance components. FDR‐corrected‐log10 *p* values for genetic (A), shared environmental (C), and unique environmental (E) covariances between 27 cerebellar measures and 9 behavioral measures. *Cry*, crystallized cognition; *dex*, dexterity; *early*, early cognition; *end*, endurance; *flu*, fluid cognition; *lan*, language task; *str*, strength; *tot*, total cognition; *WM*, working memory.

Figures S3–S8 visualize bivariate results for each variance component separately. Genetic and other variance component‐derived correlation matrices were relatively uninformative since there were many nonsignificant‐1 or 1 values owed to very small proportional variances (which obscured more accurate estimates). Therefore, the contributions to covariance components plots were more useful in revealing the underlying patterns in the data than variance correlation matrices. Cross‐cerebellar covariance was nearly entirely associated with genetic effects, while the associations between behavioral measures were split between genetic and shared environmental factors. After splitting the phenotypic covariance into components, the strongest brain‐behavioral associations were between measures of cognition and segment VI—mediated via shared environment (Figure 6). Most effects were not statistically significant after multiple testing correction. However, there were statistically significant shared environmental associations between most cerebellar ROIs and measures of total cognition, crystallized cognition, early cognition, and working memory (Figure 7), with the strongest effects seen in SPL and vermis.

## 4 Discussion

In this study, we expanded upon previous work by examining the relationships between cerebellar subvolumes and both cognitive and motor performance in a large, genetically informative sample of typically developing individuals. Our results largely align with prior evidence that cerebellar subregions play a critical role in cognitive functioning, with a comprehensive examination of cognitive measures and cerebellar subregions finding numerous small but statistically significant brain‐behavior associations. Although we hypothesized that SPL would have the strongest associations with cognition, the observed correlational patterns were less straightforward, with significant associations also seen for multiple structures in AL, IPL, and vermis. The vermis, in particular, stood out as a key player, displaying significant associations with cognitive performance across multiple measures. Previous research has highlighted the vermis as crucial to both cognition (Stoodley 2012) and motor skills (Chen et al. 2022), and our results generally concur with these findings.

The consistent significance of the vermis across all cognitive domains is notable in light of traditional views of cerebellar function, but less surprising when considering the broader literature. Although the vermis has been associated with cognitive ability in prior studies, we are unaware of any other studies that have exhaustively examined its role across a broad array of cognitive domains. Neurodevelopmental disorders demonstrate vermal cognitive roles in early development, as seen in imaging studies of young populations where structural changes—particularly volume reductions—have been linked to impairments in attention and executive function (Mackie et al. 2007; Scott et al. 2009). The structural connectivity of the vermis serves to substantiate its role in executive cognitive function. It maintains bidirectional connectivity with the medial prefrontal cortex via the cerebello‐thalamo‐cortical loop, allowing it to modulate cognitive processing (Kelly and Strick 2003).

The cerebellum may contribute to working memory by regulating cortico‐cerebellar loops that interact with executive and attentional control (Aben et al. 2012; Seese 2020). Beyond this, the vermis is functionally and structurally connected to extensive memory networks. It influences hippocampal function through a bidirectional interaction, as its projections to the anterior thalamic nuclei indirectly regulate hippocampal activity related to memory encoding and spatial navigation (Yu and Krook‐Magnuson 2015; Zeidler et al. 2020). Additionally, the vermis is linked to the amygdala, a key region for emotional and associative memory, via polysynaptic pathways (Chao et al. 2023). These anatomical and functional connections suggest that the vermis plays an important role in the coordination of working memory by integrating executive control, spatial processing, and emotional memory. The relevance of the vermis in working memory is further supported by its involvement in related disorders. For example, in Parkinson's disease, vermian atrophy has been correlated with disease progression, with more advanced structural deterioration linked to greater motor and cognitive deficits (Yin et al. 2021). Similarly, in Alzheimer's disease, alterations in vermal dendritic branching, synaptic areas, spine morphology, and structural organization suggest that the vermis may play an active role in the pathophysiology of this disease (Mavroudis et al. 2013). Finally, the vermis has also been implicated in language processing, with lexical retrieval deficits observed in cases of vermian damage (Schmahmann 2019).

Prior work has shown that individual differences in large cerebellar volumes (i.e., total cerebellar volumes, hemispheric cortical, hemispheric white matter) are strongly genetically influenced in both children and adults (Maes et al. 2023; Posthuma et al. 2000; Wallace et al. 2006). We found strong genetic effects throughout the cerebellum, particularly within the posterior lobe, findings largely consistent with prior work in a younger sample (Strike et al. 2024). Phenotypic correlations between cerebellar ROIs were nearly entirely driven by shared genetic factors. Moreover, intra‐cerebellar genetic correlations largely replicated lobar anatomy, a finding that we have previously described with cerebral ROIs (Schmitt et al. 2008). Similarly, the strongest cross‐trait genetic effects in the cerebellum are between contralateral homologues, an observation also previously described in the cerebrum (Schmitt et al. 2009). Prior genome‐wide association studies (GWAS) have identified numerous genetic variants associated with total cerebellar volumes, and loci with shared liability to schizophrenia and Alzheimer's disease (Chambers et al. 2022; Tissink et al. 2022). Our results suggest that much of the regional genetic variance is captured with a single cerebellar ROI, although there remains substantial genetically mediated regional variability that would not be captured by a global measure—and warrants further investigation. Disruptions in cerebellar structure due to genetic abnormalities may contribute to the cognitive deficits observed in conditions such as autism spectrum disorder (ASD) and schizophrenia (Jacobi et al. 2021).

Genetically‐informative bivariate models also found small but statistically significant brain‐behavioral associations, primarily with total and crystallized cognition. Interestingly, these associations appear to be primarily driven via shared environmental factors even though there were significant effects of genetic factors on individual differences in cognition, and a dominant role of additive genetic effects throughout the cerebellum. The precise environmental factors are unclear, but could potentially include in utero effects, diet, assortative mating, or gene × environmental correlation (Koeppen‐Schomerus et al. 2003; McAdams et al. 2021). In contrast, fluid intelligence had relatively weak associations with cerebellar measures.

Overall, the strength of structural associations for our motor measures was somewhat less than expected. Endurance was both moderately heritable and significantly associated with numerous ROIs throughout the cerebellum, but both Dexterity and Strength had weaker effects than hypothesized. In particular, the motor coordination required to complete the Dexterity task presumably requires cerebellar circuitry. It is possible that Dexterity‐cerebellar associations may be missed due to our focus on structural metrics. Additionally, we found that the test–retest reliability of the Dexterity measure (*r* = 0.45) was substantially lower than expected in HCP data, and measurement error may contribute to our weak findings in this domain. Other motor measures in the NIH toolbox may be more associated with cerebellar structure, for example, balance and locomotion (Reuben et al. 2013); HCP does not currently include these data, but this could represent a future research direction. Future studies could also investigate the specific genetic variants that influence cerebellar structure and function, which could provide new insights into the etiology of these disorders and potentially guide therapeutic interventions.

### 4.1 Limitations

There are several limitations of the current study which must be considered when interpreting these results. First, while the dataset is large, it is modest for quantitative genetic modeling, and the risk of type II error is higher than for tests of mean effects in a comparably sized sample. Second, the sample age range was comprised entirely of young adults, and the results may not extrapolate to younger or older populations. Third, although we included traditional covariates in our models, unmeasured confounding variables could potentially drive some of our findings (Greene et al. 2022). Reanalysis with adjustment to normative data, once available, could attenuate these effects; to our knowledge, norms are only available for our cognitive measures and only for younger subjects (Casaletto et al. 2015). To our knowledge, norms are not currently available for either our NIH Toolbox motor tasks or *CerebNet* ROIs. Third, the quantitative genetic models make the standard assumptions of the twin model, most notably that the average sharing of trait‐relevant environmental factors is assumed to be the same for MZ and DZ twins with respect to the measured phenotypes. No assortative mating is assumed, which, if present, would increase estimates of the shared environmental variance. Fourth, two of our behavioral measures were from task‐based fMRI and did not have rigorous psychometric validation. Fifth, the study focused on volumetric measures and did not examine activation patterns or cerebro‐cerebellar connectivity. A related issue is the use of discrete ROIs; prior work has shown that functional activation for specific tasks often spans multiple cerebellar lobules (Stoodley and Schmahmann 2009), and stronger brain‐behavior correlates might be identified if ROIs were defined based functionally rather than neuroanatomically.

## 5 Conclusions

This study provides further evidence of the cerebellum's role in cognition, although it also suggests that cerebellar associations are more widespread than previously described. Individual differences in cerebellar volumes are highly heritable to the lobular level. Cerebellar‐cognitive associations are weak but statistically significant and appear to be driven primarily by shared environmental factors.

## Conflicts of Interest

The authors declare no conflicts of interest.

## Supporting information


Data S1.

Data S2.

## Data Availability

The data that support the findings of this study are openly available in ConnectomeDB at https://db.humanconnectome.org.
