# Supplementary material for: The Genetics of Cerebellar Structure and Associations With Cognitive Performance: A Twin Magnetic Resonance Imaging Study
Source: Hum Brain Mapp. 2025 Aug 6;46(11):e70300. doi: 10.1002/hbm.70300 (PMC12326421; doi:10.1002/hbm.70300)
Supplement: Supplementary file 1 — Data S1. [file HBM-46-e70300-s002.docx]

**
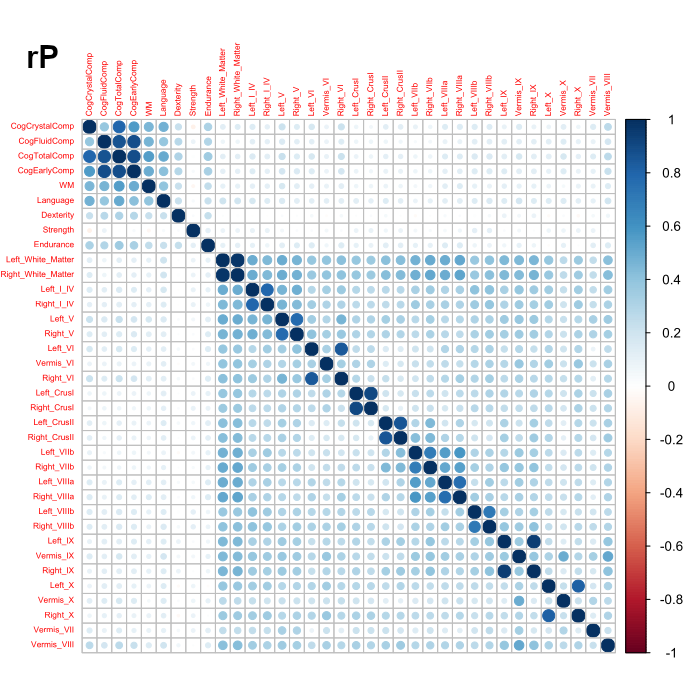
**

**Figure S1:** Phenotypic correlations, including both cognitive/motor scores and cerebellar ROIs.

**Table S1**: FDR-corrected p-values for phenotypic cerebellar-behavioral associations. Corrected values below an α of 0.05 are shown in red.

|  | **Fluid**  **Cognition** | **Crystallized**  **Cognition** | **Total**  **Cognition** | **Early**  **Cognition** | **WM**  **Task** | **Language**  **Task** | **Endurance** | **Dexterity** | **Strength** |
| --- | --- | --- | --- | --- | --- | --- | --- | --- | --- |
| **SPL** | 0.1386 | 0.0995 | 0.0481 | 0.0609 | 0.0707 | 0.0872 | 0.0001 | 0.9203 | 0.2209 |
| **IPL** | 0.0509 | 0.0995 | 0.0164 | 0.0186 | 0.0126 | 0.1319 | 0.0083 | 0.7931 | 0.4121 |
| **AL** | 0.0387 | 0.0436 | 0.0070 | 0.0139 | 0.0092 | 0.0939 | 0.0082 | 0.7931 | 0.4121 |
| **Left Cortex** | 0.0509 | 0.0325 | 0.0074 | 0.0139 | 0.0126 | 0.0635 | 0.0001 | 0.9203 | 0.2209 |
| **Right Cortex** | 0.0513 | 0.0630 | 0.0154 | 0.0201 | 0.0126 | 0.0832 | 0.0001 | 0.9062 | 0.2209 |
| **Left White** | 0.3347 | 0.8574 | 0.4142 | 0.4971 | 0.5802 | 0.2050 | 0.3404 | 0.7931 | 0.4121 |
| **Right White** | 0.2908 | 0.9900 | 0.4315 | 0.4971 | 0.5772 | 0.3162 | 0.1488 | 0.7931 | 0.2629 |
| **Vermis** | 0.0076 | 0.0000 | 0.0000 | 0.0007 | 0.0092 | 0.0148 | 0.0007 | 0.8747 | 0.6924 |
| **Left_CrusII** | 0.4157 | 0.1150 | 0.1338 | 0.2573 | 0.2485 | 0.0832 | 0.0025 | 0.7931 | 0.4121 |
| **Left_I_IV** | 0.1133 | 0.1065 | 0.0367 | 0.0609 | 0.1015 | 0.0832 | 0.0857 | 0.7931 | 0.2209 |
| **Left_IX** | 0.0513 | 0.7000 | 0.1067 | 0.0254 | 0.0822 | 0.1717 | 0.5419 | 0.6160 | 0.8604 |
| **Left_V** | 0.0287 | 0.0065 | 0.0013 | 0.0105 | 0.0065 | 0.2004 | 0.0090 | 0.9970 | 0.8118 |
| **Left_VI** | 0.0610 | 0.0153 | 0.0070 | 0.0201 | 0.0822 | 0.2918 | 0.0010 | 0.7931 | 0.8922 |
| **Left_VIIb** | 0.4555 | 0.8574 | 0.7823 | 0.9862 | 0.9051 | 0.8212 | 0.0902 | 0.7931 | 0.6924 |
| **Left_VIIIa** | 0.4413 | 0.2547 | 0.1832 | 0.3279 | 0.0839 | 0.3954 | 0.0040 | 0.9378 | 0.8604 |
| **Left_VIIIb** | 0.0610 | 0.0630 | 0.0211 | 0.0253 | 0.1559 | 0.2422 | 0.4857 | 0.7931 | 0.1355 |
| **Left_X** | 0.0513 | 0.7633 | 0.1067 | 0.1942 | 0.2658 | 0.0914 | 0.1432 | 0.7931 | 0.3547 |
| **Right_CrusI** | 0.5054 | 0.7633 | 0.8979 | 0.7832 | 0.3238 | 0.2394 | 0.0832 | 0.9760 | 0.2209 |
| **Right_CrusII** | 0.6090 | 0.1939 | 0.2438 | 0.3279 | 0.3238 | 0.1641 | 0.0082 | 0.9760 | 0.2209 |
| **Right_I_IV** | 0.1499 | 0.2934 | 0.1067 | 0.2232 | 0.0757 | 0.1641 | 0.3465 | 0.7931 | 0.2209 |
| **Right_IX** | 0.0851 | 0.7245 | 0.1404 | 0.0609 | 0.0880 | 0.2004 | 0.3370 | 0.6160 | 0.8604 |
| **Right_V** | 0.1287 | 0.2789 | 0.0977 | 0.0253 | 0.0597 | 0.3162 | 0.0039 | 0.9062 | 0.8922 |
| **Right_VI** | 0.0287 | 0.0004 | 0.0003 | 0.0047 | 0.0328 | 0.3896 | 0.0014 | 0.7931 | 0.8922 |
| **Right_VIIb** | 0.8977 | 0.9032 | 0.8979 | 0.9862 | 0.7483 | 0.5738 | 0.0903 | 0.9548 | 0.9896 |
| **Right_VIIIa** | 0.3668 | 0.1065 | 0.1223 | 0.2616 | 0.0328 | 0.3908 | 0.0014 | 0.9406 | 0.6924 |
| **Right_VIIIb** | 0.0851 | 0.2547 | 0.0797 | 0.0609 | 0.3067 | 0.1717 | 0.3465 | 0.7931 | 0.1355 |
| **Right_X** | 0.1386 | 0.9032 | 0.2438 | 0.3357 | 0.1559 | 0.2516 | 0.5699 | 0.7931 | 0.2209 |
| **Vermis_IX** | 0.1687 | 0.2293 | 0.0797 | 0.1942 | 0.2453 | 0.1175 | 0.0918 | 0.7931 | 0.8604 |
| **Vermis_VI** | 0.3380 | 0.4744 | 0.2540 | 0.3279 | 0.0880 | 0.1717 | 0.3404 | 0.7931 | 0.6924 |
| **Vermis_VII** | 0.1687 | 0.0067 | 0.0164 | 0.1138 | 0.0892 | 0.2516 | 0.0059 | 0.7931 | 0.2209 |
| **Vermis_VIII** | 0.0026 | 0.0000 | 0.0000 | 0.0000 | 0.0092 | 0.0148 | 0.0006 | 0.7931 | 0.7453 |
| **Vermis_X** | 0.0287 | 0.1058 | 0.0074 | 0.0146 | 0.8008 | 0.0850 | 0.0638 | 0.9406 | 0.3440 |

**Table S2:** Standardized beta weights for cerebellar ROI volumes influence on cognitive and motor measures (after controlling for age, sex, and TBV).

|  | Fluid Cognition | Crystallized Cognition | Total  Cognition | Early Cognition | WM  Task | Language  Task | Endurance | Dexterity | Strength |
| --- | --- | --- | --- | --- | --- | --- | --- | --- | --- |
| **AL** | 0.1149 | 0.1074 | 0.1369 | 0.1295 | 0.1425 | 0.0919 | 0.1181 | -0.0329 | 0.0329 |
| **SPL** | 0.0675 | 0.0844 | 0.0909 | 0.0862 | 0.0899 | 0.0905 | 0.1755 | 0.0119 | 0.0506 |
| **IPL** | 0.1219 | 0.0938 | 0.1301 | 0.1353 | 0.1413 | 0.0946 | 0.1356 | 0.0566 | 0.0416 |
| **Left Cortex** | 0.1121 | 0.1186 | 0.1393 | 0.1354 | 0.1383 | 0.1221 | 0.2002 | 0.0164 | 0.0556 |
| **Right Cortex** | 0.1066 | 0.1049 | 0.1273 | 0.1242 | 0.1358 | 0.1080 | 0.1910 | 0.0192 | 0.0614 |
| **Left White Matter** | -0.0546 | -0.0101 | -0.0400 | -0.0346 | 0.0305 | 0.0654 | 0.0425 | -0.0333 | 0.0324 |
| **Right White Matter** | -0.0601 | 0.0018 | -0.0376 | -0.0361 | 0.0336 | 0.0470 | 0.0642 | -0.0368 | 0.0438 |
| **Vermis** | 0.1503 | 0.1980 | 0.2072 | 0.1734 | 0.1352 | 0.1429 | 0.1573 | 0.0228 | 0.0243 |
| **Left_I_IV** | 0.0788 | 0.0761 | 0.0980 | 0.0872 | 0.0795 | 0.1004 | 0.0757 | -0.0401 | 0.0471 |
| **Right_I_IV** | 0.0692 | 0.0482 | 0.0748 | 0.0587 | 0.0897 | 0.0722 | 0.0391 | -0.0381 | 0.0512 |
| **Left_V** | 0.1131 | 0.1288 | 0.1445 | 0.1237 | 0.1449 | 0.0588 | 0.1023 | -0.0032 | 0.0116 |
| **Right_V** | 0.0678 | 0.0512 | 0.0738 | 0.0984 | 0.0914 | 0.0413 | 0.1157 | -0.0194 | -0.0067 |
| **Left_VI** | 0.0789 | 0.1024 | 0.1122 | 0.0957 | 0.0734 | 0.0425 | 0.1247 | 0.0281 | -0.0034 |
| **Vermis_VI** | 0.0399 | 0.0322 | 0.0428 | 0.0397 | 0.0700 | 0.0600 | 0.0379 | -0.0431 | 0.0200 |
| **Right_VI** | 0.0997 | 0.1439 | 0.1480 | 0.1245 | 0.0934 | 0.0332 | 0.1191 | 0.0271 | -0.0064 |
| **Left_CrusI** | 0.0287 | -0.0044 | 0.0120 | 0.0251 | 0.0445 | 0.0520 | 0.0746 | -0.0116 | 0.0439 |
| **Right_CrusI** | 0.0247 | -0.0147 | 0.0047 | 0.0125 | 0.0394 | 0.0521 | 0.0699 | 0.0015 | 0.0540 |
| **Left_CrusII** | 0.0290 | 0.0640 | 0.0556 | 0.0445 | 0.0454 | 0.0814 | 0.1076 | 0.0345 | 0.0286 |
| **Right_CrusII** | 0.0177 | 0.0537 | 0.0422 | 0.0374 | 0.0361 | 0.0600 | 0.0953 | -0.0029 | 0.0425 |
| **Left_VIIb** | -0.0325 | 0.0062 | -0.0167 | -0.0005 | -0.0050 | -0.0111 | 0.0718 | -0.0524 | 0.0207 |
| **Right_VIIb** | -0.0056 | -0.0084 | -0.0081 | -0.0017 | 0.0147 | 0.0196 | 0.0681 | -0.0052 | 0.0005 |
| **Left_VIIIa** | 0.0359 | 0.0521 | 0.0572 | 0.0445 | 0.0818 | 0.0347 | 0.1264 | 0.0145 | 0.0129 |
| **Right_VIIIa** | 0.0423 | 0.0791 | 0.0704 | 0.0546 | 0.1099 | 0.0362 | 0.1480 | 0.0094 | 0.0233 |
| **Left_VIIIb** | 0.0975 | 0.0914 | 0.1093 | 0.1101 | 0.0693 | 0.0573 | 0.0319 | -0.0313 | 0.0729 |
| **Right_VIIIb** | 0.0872 | 0.0574 | 0.0851 | 0.0915 | 0.0486 | 0.0716 | 0.0415 | 0.0286 | 0.0712 |
| **Left_IX** | 0.0884 | 0.0204 | 0.0653 | 0.0932 | 0.0744 | 0.0628 | 0.0234 | 0.0769 | -0.0056 |
| **Vermis_IX** | 0.0616 | 0.0576 | 0.0800 | 0.0612 | 0.0561 | 0.0789 | 0.0687 | 0.0444 | 0.0070 |
| **Right_IX** | 0.0771 | 0.0188 | 0.0591 | 0.0803 | 0.0725 | 0.0579 | 0.0405 | 0.0786 | -0.0075 |
| **Left_X** | 0.0833 | 0.0173 | 0.0644 | 0.0549 | 0.0448 | 0.0776 | -0.0566 | -0.0445 | 0.0340 |
| **Vermis_X** | 0.0989 | 0.0693 | 0.1066 | 0.1009 | -0.0079 | 0.0789 | 0.0686 | 0.0059 | 0.0313 |
| **Right_X** | 0.0586 | 0.0077 | 0.0441 | 0.0374 | 0.0557 | 0.0451 | -0.0206 | -0.0615 | 0.0461 |
| **Vermis_VII** | 0.0548 | 0.1106 | 0.0956 | 0.0637 | 0.0647 | 0.0456 | 0.1001 | 0.0326 | 0.0480 |
| **Vermis_VIII** | 0.1625 | 0.2360 | 0.2341 | 0.2008 | 0.1264 | 0.1325 | 0.1525 | 0.0474 | -0.0155 |

**
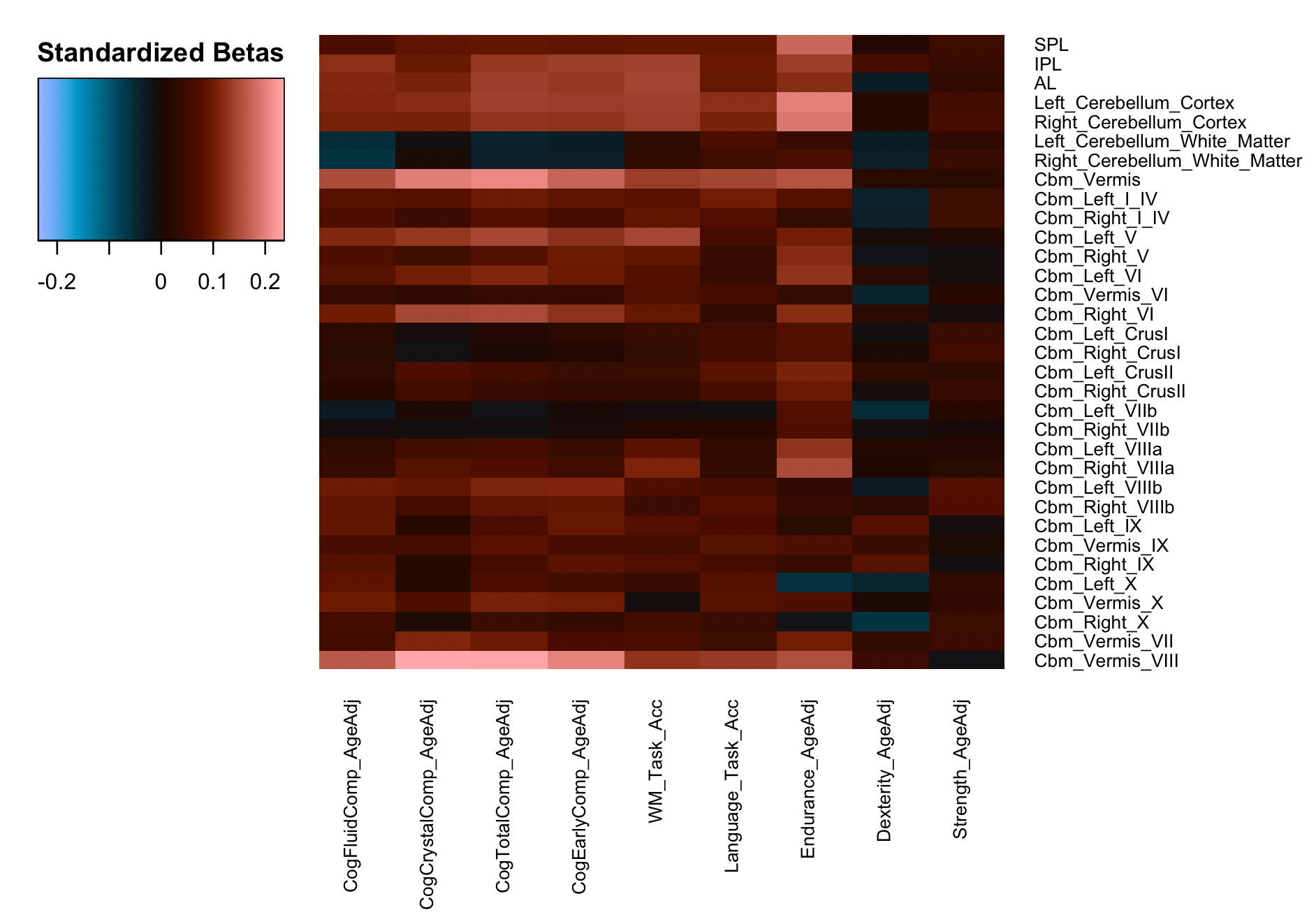
**

**Figure S2:** Standardized β weights for phenotypic regression models.

**Table S3**: Maximum Likelihood Estimates for Cerebellar Measures, including 95% Confidence Intervals (no TBV covariate).

| **ROI** | $\hat{a}^{2}$ |  | $\hat{c}^{2}$ |  | $\hat{e}^{2}$ |  |
| --- | --- | --- | --- | --- | --- | --- |
| Left_Cerebellum_Cortex | 0.84 | [0.68 0.90] | 0.04 | [0.00 0.19] | 0.13 | [0.10 0.17] |
| Left_Cerebellum_White_Matter | 0.79 | [0.72 0.84] | 0.00 | [0.00 0.12] | 0.21 | [0.16 0.28] |
| Right_Cerebellum_White_Matter | 0.81 | [0.74 0.85] | 0.00 | [0.00 0.11] | 0.19 | [0.15 0.26] |
| Right_Cerebellum_Cortex | 0.78 | [0.63 0.88] | 0.06 | [0.00 0.20] | 0.16 | [0.12 0.21] |
| Cbm_Left_I_IV | 0.62 | [0.39 0.70] | 0.00 | [0.00 0.16] | 0.38 | [0.30 0.48] |
| Cbm_Right_I_IV | 0.63 | [0.40 0.73] | 0.02 | [0.00 0.20] | 0.35 | [0.27 0.44] |
| Cbm_Left_V | 0.47 | [0.22 0.68] | 0.13 | [0.00 0.30] | 0.40 | [0.31 0.52] |
| Cbm_Right_V | 0.72 | [0.59 0.78] | 0.00 | [0.00 0.09] | 0.28 | [0.22 0.37] |
| Cbm_Left_VI | 0.79 | [0.61 0.85] | 0.02 | [0.00 0.17] | 0.20 | [0.15 0.26] |
| Cbm_Vermis_VI | 0.46 | [0.26 0.65] | 0.23 | [0.07 0.38] | 0.31 | [0.24 0.40] |
| Cbm_Right_VI | 0.74 | [0.57 0.85] | 0.07 | [0.00 0.22] | 0.19 | [0.15 0.26] |
| Cbm_Left_CrusI | 0.86 | [0.74 0.89] | 0.00 | [0.00 0.14] | 0.14 | [0.11 0.18] |
| Cbm_Right_CrusI | 0.81 | [0.75 0.85] | 0.00 | [0.00 0.19] | 0.19 | [0.15 0.25] |
| Cbm_Left_CrusII | 0.73 | [0.51 0.79] | 0.00 | [0.00 0.18] | 0.27 | [0.21 0.35] |
| Cbm_Right_CrusII | 0.73 | [0.65 0.79] | 0.00 | [0.00 0.11] | 0.27 | [0.21 0.35] |
| Cbm_Left_VIIb | 0.56 | [0.41 0.66] | 0.00 | [0.00 0.10] | 0.44 | [0.34 0.55] |
| Cbm_Right_VIIb | 0.57 | [0.46 0.66] | 0.00 | [0.00 0.08] | 0.43 | [0.34 0.54] |
| Cbm_Left_VIIIa | 0.70 | [0.61 0.77] | 0.00 | [0.00 0.09] | 0.30 | [0.23 0.39] |
| Cbm_Right_VIIIa | 0.71 | [0.62 0.78] | 0.00 | [0.00 0.10] | 0.29 | [0.22 0.38] |
| Cbm_Left_VIIIb | 0.65 | [0.42 0.73] | 0.00 | [0.00 0.11] | 0.35 | [0.27 0.44] |
| Cbm_Right_VIIIb | 0.53 | [0.27 0.70] | 0.09 | [0.00 0.28] | 0.38 | [0.29 0.50] |
| Cbm_Left_IX | 0.83 | [0.77 0.87] | 0.00 | [0.00 0.12] | 0.17 | [0.13 0.23] |
| Cbm_Vermis_IX | 0.70 | [0.50 0.80] | 0.04 | [0.00 0.20] | 0.26 | [0.20 0.34] |
| Cbm_Right_IX | 0.79 | [0.62 0.84] | 0.00 | [0.00 0.02] | 0.21 | [0.16 0.27] |
| Cbm_Left_X | 0.71 | [0.51 0.80] | 0.03 | [0.00 0.19] | 0.25 | [0.20 0.34] |
| Cbm_Vermis_X | 0.63 | [0.42 0.70] | 0.00 | [0.00 0.10] | 0.37 | [0.30 0.47] |
| Cbm_Right_X | 0.55 | [0.33 0.75] | 0.14 | [0.00 0.31] | 0.30 | [0.23 0.40] |
| Cbm_Vermis_VII | 0.74 | [0.66 0.80] | 0.00 | [0.00 0.09] | 0.26 | [0.20 0.34] |
| Cbm_Vermis_VIII | 0.73 | [0.56 0.84] | 0.07 | [0.00 0.22] | 0.20 | [0.15 0.26] |
| Cbm_Vermis | 0.64 | [0.47 0.81] | 0.16 | [0.00 0.30] | 0.20 | [0.16 0.26] |

**Table S4**: Maximum Likelihood Estimates for Cerebellar Measures, including 95% Confidence Intervals (with TBV covariate).

| **ROI** | $\hat{a}^{2}$ |  | $\hat{c}^{2}$ |  | $\hat{e}^{2}$ |  |
| --- | --- | --- | --- | --- | --- | --- |
| Left_Cerebellum_Cortex | 0.84 | [0.60 0.87] | 0.04 | [0.00 0.23] | 0.13 | [0.12 0.21] |
| Left_Cerebellum_White_Matter | 0.79 | [0.66 0.80] | 0.00 | [0.00 0.03] | 0.21 | [0.20 0.34] |
| Right_Cerebellum_White_Matter | 0.81 | [0.69 0.82] | 0.00 | [0.00 0.09] | 0.19 | [0.18 0.31] |
| Right_Cerebellum_Cortex | 0.78 | [0.57 0.86] | 0.06 | [0.00 0.25] | 0.16 | [0.13 0.22] |
| Cbm_Left_I_IV | 0.62 | [0.32 0.66] | 0.00 | [0.00 0.01] | 0.38 | [0.34 0.54] |
| Cbm_Right_I_IV | 0.63 | [0.33 0.70] | 0.02 | [0.00 0.22] | 0.35 | [0.30 0.48] |
| Cbm_Left_V | 0.47 | [0.17 0.64] | 0.13 | [0.00 0.29] | 0.40 | [0.36 0.58] |
| Cbm_Right_V | 0.72 | [0.58 0.76] | 0.00 | [0.00 0.09] | 0.28 | [0.24 0.42] |
| Cbm_Left_VI | 0.79 | [0.56 0.83] | 0.02 | [0.00 0.19] | 0.20 | [0.18 0.30] |
| Cbm_Vermis_VI | 0.46 | [0.25 0.64] | 0.23 | [0.08 0.39] | 0.31 | [0.24 0.40] |
| Cbm_Right_VI | 0.74 | [0.58 0.85] | 0.07 | [0.00 0.20] | 0.19 | [0.15 0.26] |
| Cbm_Left_CrusI | 0.86 | [0.78 0.87] | 0.00 | [0.00 0.05] | 0.14 | [0.13 0.22] |
| Cbm_Right_CrusI | 0.81 | [0.57 0.84] | 0.00 | [0.00 0.20] | 0.19 | [0.16 0.27] |
| Cbm_Left_CrusII | 0.73 | [0.55 0.79] | 0.00 | [0.00 0.10] | 0.27 | [0.21 0.34] |
| Cbm_Right_CrusII | 0.73 | [0.65 0.79] | 0.00 | [0.00 0.09] | 0.27 | [0.21 0.35] |
| Cbm_Left_VIIb | 0.56 | [0.41 0.62] | 0.00 | [0.00 0.12] | 0.44 | [0.38 0.59] |
| Cbm_Right_VIIb | 0.57 | [0.44 0.64] | 0.00 | [0.00 0.09] | 0.43 | [0.36 0.56] |
| Cbm_Left_VIIIa | 0.70 | [0.57 0.74] | 0.00 | [0.00 0.11] | 0.30 | [0.26 0.43] |
| Cbm_Right_VIIIa | 0.71 | [0.51 0.75] | 0.00 | [0.00 0.05] | 0.29 | [0.25 0.42] |
| Cbm_Left_VIIIb | 0.65 | [0.41 0.69] | 0.00 | [0.00 0.04] | 0.35 | [0.31 0.49] |
| Cbm_Right_VIIIb | 0.53 | [0.25 0.68] | 0.09 | [0.00 0.26] | 0.38 | [0.32 0.53] |
| Cbm_Left_IX | 0.83 | [0.63 0.86] | 0.00 | [0.00 0.17] | 0.17 | [0.14 0.23] |
| Cbm_Vermis_IX | 0.70 | [0.50 0.75] | 0.04 | [0.00 0.11] | 0.26 | [0.25 0.41] |
| Cbm_Right_IX | 0.79 | [0.57 0.84] | 0.00 | [0.00 0.20] | 0.21 | [0.16 0.28] |
| Cbm_Left_X | 0.71 | [0.48 0.79] | 0.03 | [0.00 0.21] | 0.25 | [0.21 0.36] |
| Cbm_Vermis_X | 0.63 | [0.36 0.65] | 0.00 | [0.00 0.09] | 0.37 | [0.35 0.54] |
| Cbm_Right_X | 0.55 | [0.20 0.66] | 0.14 | [0.05 0.38] | 0.30 | [0.25 0.45] |
| Cbm_Vermis_VII | 0.74 | [0.55 0.78] | 0.00 | [0.00 0.09] | 0.26 | [0.22 0.37] |
| Cbm_Vermis_VIII | 0.73 | [0.58 0.82] | 0.07 | [0.00 0.17] | 0.20 | [0.18 0.30] |
| Cbm_Vermis | 0.64 | [0.45 0.80] | 0.16 | [0.00 0.27] | 0.20 | [0.19 0.32] |


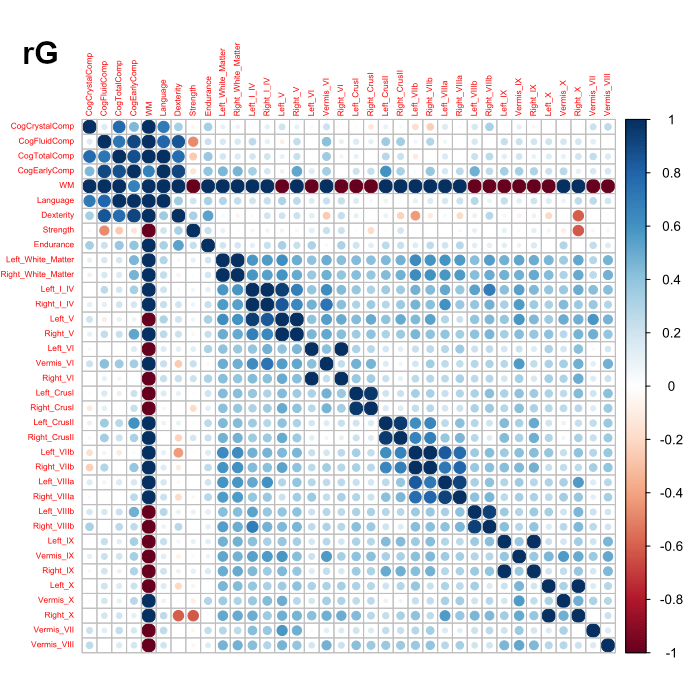


**Figure S3:** Genetic correlations, including cognitive scores and cerebellar ROIs.

**
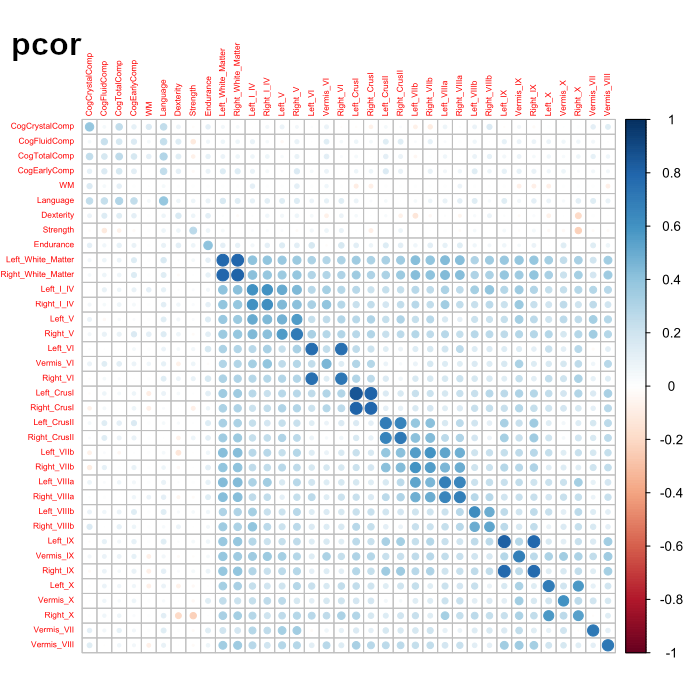
Figure S4:** Contributions of genetic effects to phenotypic covariance. Additive genetic proportional variances (i.e. the heritability) is shown along the diagonal.

**
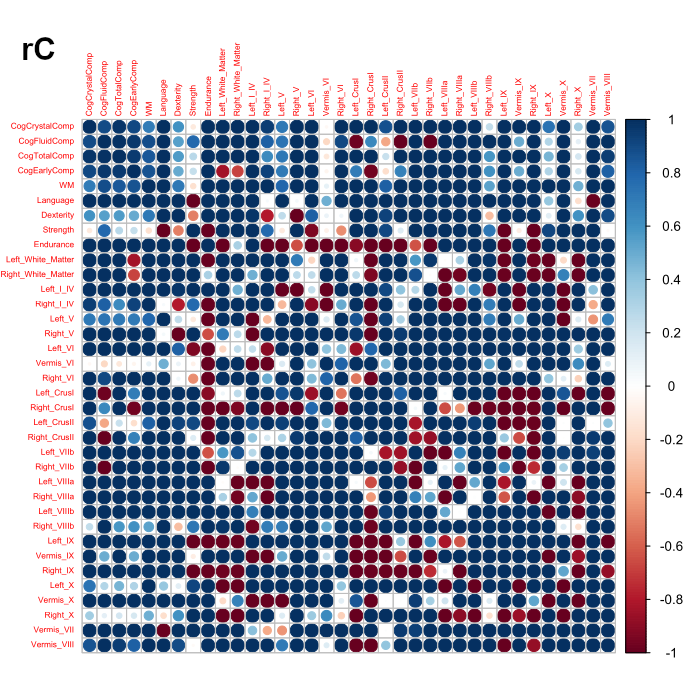
Figure S5**: Shared environmental correlations for brain and behavioral measures.

**
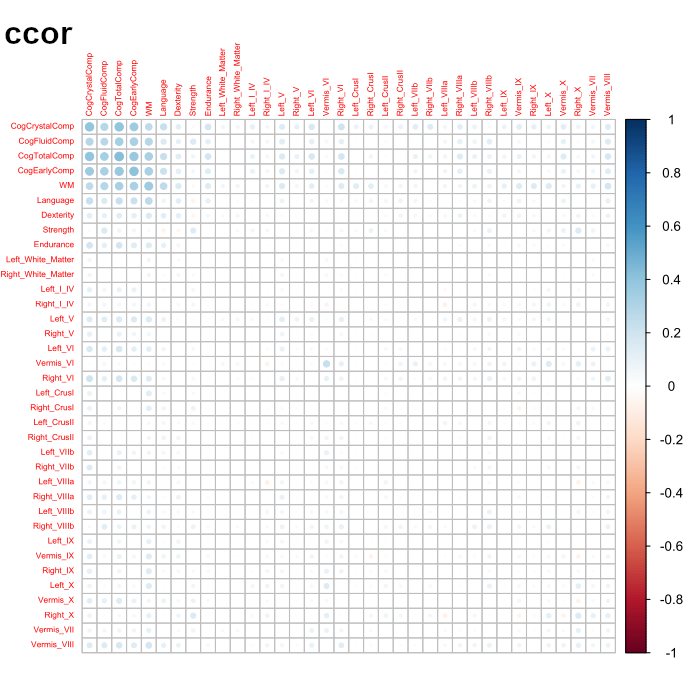
Figure S6**: Contributions of shared environmental factors to phenotypic covariance for brain and behavioral measures.

**
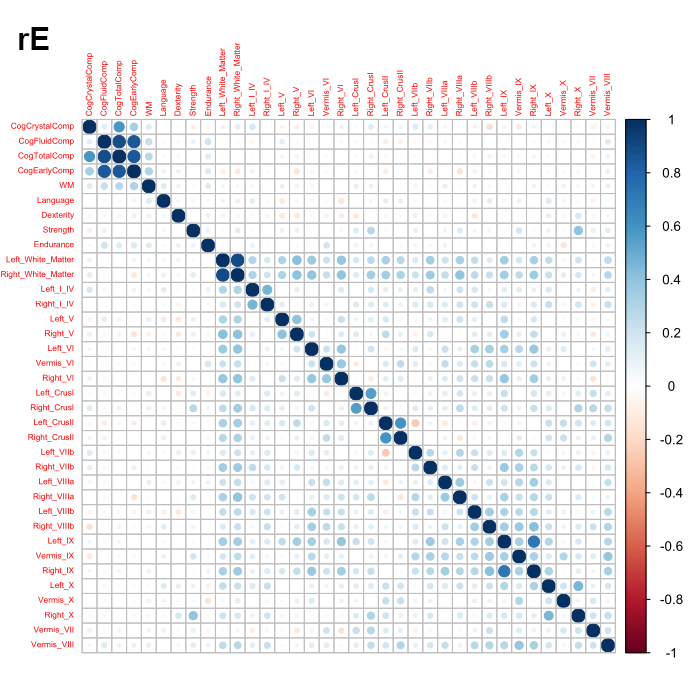
Figure S7:** Unique environmental correlations.

**
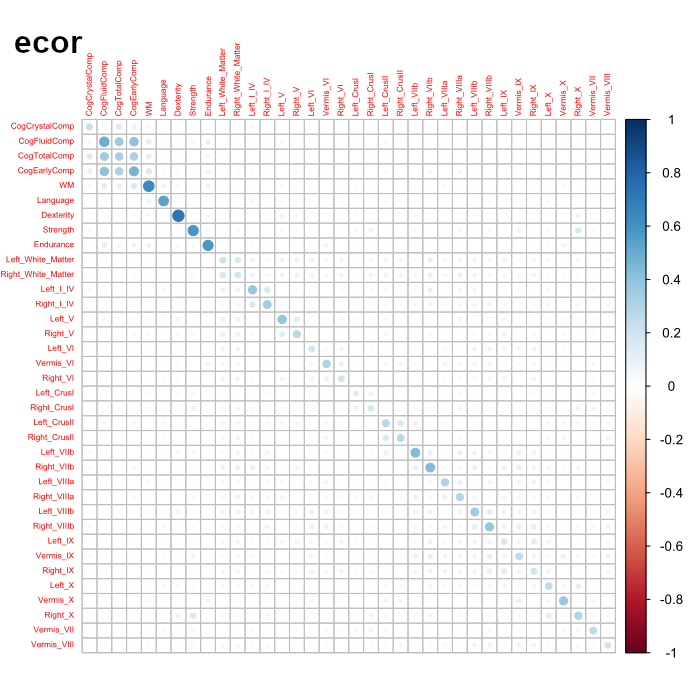
Figure S8:** Contributions of unique environmental factors to phenotypic covariance for brain and behavioral measures.
